# Simple and rapid assay for effect of the new oral anticoagulant (NOAC) rivaroxaban: preliminary results support further tests with all NOACs

**DOI:** 10.1186/1477-9560-12-7

**Published:** 2014-03-21

**Authors:** Raul Altman, Claudio Daniel Gonzalez

**Affiliations:** 1 Centro de Trombosis de Buenos Aires, Viamonte 2008, Buenos Aires 1056, Argentina; 2 Catedra de Farmacologia, Facultad de Medicina, Universidad Nacional de Buenos Aires, Viamonte 2008, Buenos Aires 1056, Argentina

**Keywords:** NOACs, Oral anticoagulant, Rivaroxaban, Anti-factor X activity, DVT, Atrial fibrillation

## Abstract

**Background:**

New oral anticoagulant (NOAC) drugs are known to influence the results of some routine hemostasis tests. Further data are needed to enable routine assessment of the effects of NOAC on clotting parameters in some special circumstances.

**Methods:**

Following administration of rivaroxaban to patients, at the likely peak and trough activity times, we assessed the effects on prothrombin time (PT), activated partial thromboplastin time (APTT), thrombin time (TT), and clotting time using Russell’s viper venom, and in the presence of phospholipids and calcium reagent available as DVVreagent® and DVVconfirm®. The data were used to determine an adequate NOAC plasma level based on anticoagulant activities expressed as a ratio (patients/normal, R-C).

**Results:**

DVVconfirm as R-C could be rapidly performed, and the results were reasonably sensitive for rivaroxaban and probably for other FX inhibitors. This assay is not influenced by lupus anticoagulant and heparin, does not require purified NOAC as control, and will measure whole-plasma clotting activity.

**Conclusions:**

We propose a cut-off R-C value of 4.52 ± 0.33 for safety, but clinical studies are needed to establish whether this cut-off is useful for identifying patients at increased risk of hemorrhage or exhibiting low anticoagulation effect. It also seems possible that normal R-C could indicate an absence of anticoagulant activity when rivaroxaban is discontinued due to episodes of uncontrolled bleeding during anticoagulation or for emergency surgery.

## Background

Anticoagulant therapy has well-established benefits, but also carries the risk of major or fatal bleeding complications. Furthermore, the beneficial effects of anticoagulant therapy are sometimes inadequate, in which case the loss of protection favors the development of thromboembolic complications [1]. As alternatives to heparin (and its congeners) and vitamin K antagonists (VKAs), novel oral anticoagulants (NOACs) have been developed for use in preventing venous thrombosis in surgical patients, or stroke and systemic embolism (SE) in patients with atrial fibrillation (AF) [2-8]. Several phase 3 trials have shown that NOACs are at least non-inferior to low-molecular-weight heparin and to VKAs, but their clear superiority in terms of overall and vascular mortality has not consistently been proven.

It has been postulated that NOACs can be given at fixed doses with no monitoring requirements; however, some clinical circumstances will still require measurement of the anticoagulant effect of a NOAC [9,10]. Combining antiplatelet drugs and warfarin increases the bleeding risk [11-14]. In situations requiring warfarin and dual antiplatelet therapy, it is proposed that the level of anticoagulation should be lowered to an international normalized ratio (INR) of 2.0–2.5 to reduce bleeding risks without affecting therapeutic efficacy [15]. Moreover, investigations of patients taking two or three medications, including a NOAC, have shown significant increases in major bleeding [16-18]. Under circumstances where NOACs may increase the risk of bleeding to the extent that it exceeds the benefits, it would be valuable to have a simple coagulation assay to accurately measure the drug effect [19,20].

In contrast to warfarin, the anticoagulant effect of NOACs is difficult to measure. A variety of routine coagulation assays are recommended for assessing drug effects; however, these tests are not readily available to most clinical laboratories. Additionally, in vitro study results cannot be extrapolated to patients due to variable drug concentrations in the elderly, dependence on renal function, concomitant use of P-glycoprotein activators or inhibitors, adherence to treatment, and pharmacodynamic parameters [21-24]. Studies demonstrate poor correlations between drug effects and assay results, and only limited data are available regarding the sensitivities of different reagent/instrument combinations [25-31].

This prospective study was designed to investigate the use of different commercially available reagents for measuring NOAC coagulation and pharmacodynamic effects, and to develop an assay for oral anti-factor Xa drugs. The reagent used in the current study contains Russell’s viper venom, which directly activates factor X to Factor Xa in the presence of phospholipids and calcium, and is available as DVVreagent® and DVVconfirm®.

## Methods

### Normal donors

Twenty-five healthy volunteers (13 women and 12 men) with no history of thromboembolic or hemorrhagic diseases, cardiac, renal, hepatic, or malignant diseases, were required to be drug free for 10 days before the study. Only platelet poor plasma from subjects with a normal prothrombin time, activated partial thromboplastin time and thromnin time that fulfilled the inclusion criteria were used.

### Patients

This study included 170 patients receiving prolonged oral VKA therapy (warfarin or acenocoumarol) who were referred to our thrombosis center for follow-up anticoagulant therapy. Data from this patient group were used to create a reference curve, for comparison with the DVVconfirm® rate.

The study also included 25 patients who were treated with the new oral anticoagulant rivaroxaban (15 men, 10 women; mean age, 66.7 ± 16.4 years; median age, 70 years). These patients had no history of hemorrhagic diseases, and were referred to our clinic after their own physician recommended drug use due to deep venous thrombosis (9 patients), portal vein thrombosis (1 patient), atrial fibrillation (14 patients), or coronary stenting (1 patient). All patients were treated with 20 mg rivaroxaban per day, except for one woman who received 15 mg daily. Medication was taken at the same hour each morning. Double antithrombotic (rivaroxaban and aspirin) therapy was indicated in four patients.

### Additional medical therapy

Most patients were receiving additional medications at the time of the study, some of which could influence NOAC metabolism [22,23], including antidepressants (n = 8), antidiabetics (n = 1), anxiolytics (n = 10), beta-blockers (n = 8), ACE inhibitors (n = 8), lipid-lowering agents (n = 11), protein pump inhibitor (PPI) (n = 6), levothyroxine (n = 3), diuretics (n = 5), aspirin (n = 4), non-steroidal anti-inflammatory (n = 2), lanoxin (n = 1), allopurinol (n = 2), amiodarone (n = 4), and medication for prostatic hypertrophy (n = 2). No patients were active smokers. Blood hepatic enzymes and creatinine were assayed. Serum creatinine levels were below 1.2 mg/mL in all patients, except one patient who had a creatinine level of 1.4 mg/mL and a creatinine clearance of 66 mg/minutes. Patients had to be receiving therapy for ≥7 days before entering the study.

### Hemostasis tests

Venous blood was drawn from the antecubital vein without stasis, and mixed with 0.11 mol/L sodium citrate (1:10 v/v). Samples were obtained in the morning for the following hemostasis studies: prothrombin time (PT), activated partial thromboplastin time (APTT), thrombin time (TT), and DVV reagent to perform the DVV test and DVVconfirm® time. The INR reference curve was created using data from patients with stable INRs and values within the therapeutic range (2.0–3.5). Samples were not included if a patient reported being off the assigned therapy within the prior six weeks. For INR 1.2–1.99 or >3.5, this rule was not applied, and all samples were incorporated into the analysis.

From the patients taking rivaroxaban, samples were obtained at the time of peak drug activity after drug ingestion (mean, 2.06 ± 0.29 h; median, 2.1 h), and during the activity trough at 0–2 hours before the next dose (mean, 23.4 ± 1.09 h; median, 23.5 h). None of the plasma samples required hematocrit adjustment. Tests were performed within 3 h of sampling.

Platelet-poor plasma (PPP) was obtained by centrifuging blood samples at 900 × *g* for 15 min. Plastic syringes, tubes, and pipettes were used for all tests. PT, DVVtest®, and DVVconfirm® were measured in citrated PPP samples using a coagulometer ST4 (Diagnostica Stago, Asnieres, France). Prothrombin time was determined using rabbit brain thromboplastin Neoplastin Plus® (International Sensitivity Index (ISI) 1.31; Diagnostica Stago, Asnières-sur-Seine, France). DVVtest® and DVVconfirm® were performed using the reagents from Sekisui Diagnostics (Stanford, USA) following the manufacturer’s indications. aPTT was measured using TriniClot aPTT HS reagent (Trinity Biotech, Ireland) with manual methods at 37°C, according to the manufacturer’s instructions.

Thrombin time was measured using a lyophilized preparation containing bovine thrombin (~75 NIH U/mL), which was included in the TrinitCLOT™ Fibrinogen kit (Trinity Biotech, Ireland). This powder was diluted following the manufacturer’s instructions, and then diluted 1:10 with distilled water to create the working solution. The diluted thrombin reagent (100 μL) was then preincubated at 37°C and mixed with 100 μL normal or patient PPP. Normal PPP clotted at around 20 sec, which was compared with patient plasma.

For comparison between PT, aPTT, TT, DVVtest, and DVVconfirm, the time coefficients of patients/normal values were obtained and each expressed as a ratio (R-PT, R-aPTT, R-TT, R-VV, and R-C, respectively). The means from the 25 normal donors were used as normal values. Samples were obtained over three consecutive days, and were prepared using techniques similar to those used for preparing patient blood samples. The samples were tested within three hours of sampling. Reported results are the means of duplicate assays. Triniclot Factor X (Trinity Biotech, Ireland) was used as Factor X-deficient plasma. The lyophilized product was dissolved in distilled water.

### Statistical analysis

Quantitative variables are expressed as mean ± SD, and differences between quantitative data were evaluated by analysis of variance (ANOVA). Qualitative data are presented as percentages, and associations between qualitative data were examined using the Yates-corrected chi-squared test. Among the patients treated with VKAs, the correlation between confirm and PT was assessed using the Pearson method (simple linear model) to obtain the Pearson r coefficient. The agreement between DVVconfirm and PT was examined by plotting the difference between these values against the average of the two values, following the Bland-Altman technique. In patients treated with rivaroxaban, the differences between DVVconfirm and other in vitro tests (PT, aPTT, TT, and DVVtest) were assessed at peak and trough by applying ANOVA and Scheffe post-hoc tests. Statistical significance was accepted when p values were below 0.05.

## Results

One reference curve was obtained by testing DVVconfirm in 170 patients receiving stable anticoagulant therapy with coumadins. The INR a suitable system for minimizing the reagent variability of different thromboplastins [32] was used for comparison to evaluate the intensity of anticoagulation. Ratios were calculated (patients/normal, both in seconds) to determine correlations between the ratio of INR curves of patients treated with coumadin (R-PT) and the ratio with DVVconfirm® (R-C). A second reference curve was obtained by testing a mixture of normal plasma and factor X-deficient plasma to obtain factor X concentrations of between 100% and 1.56%. Then PT, aPTT, DVVtest, and DVVconfirm times were recorded.

### Correlation between R-PT and R-C

**Figure 1:**
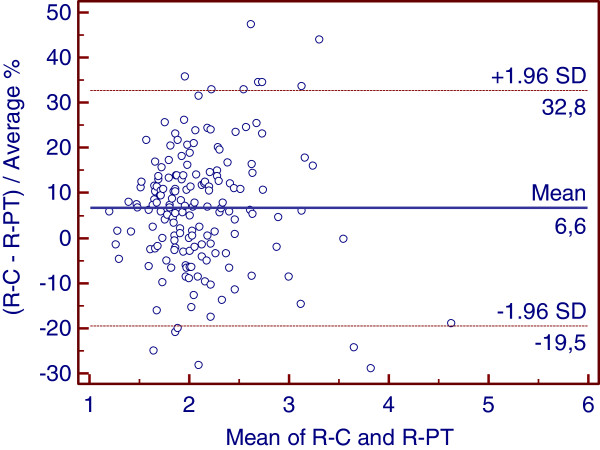
**Agreement between R-PT and R-C in 170 patients treated with warfarin.** (R-PT – RC)/Average% plotted against the mean of R-PT and RC.

Warfarin affects the levels of plasma vitamin K-dependent clotting proteins (indicated by R-PT), and rivaroxaban inhibits only factor X (indicated by R-C); thus, a direct relationship between R-PT and R-C is unlikely. Among the group of 170 patients, the INR varied between 1.22 and 8.38, with R-PT of 1.22 and 5.07, respectively, and DVVconfirm R-C values between 1.24 and 4.19. Eleven patients showed INR values of 3.51–8.38 (R-PT, 2.74–5.07), corresponding to DVVconfirm R-C of 2.95–4.19. Thirty-six patients had INR values of 1.22–1.99 (R-PT, 1.17–1.67), corresponding to DVVconfirm R-C of 1.24–1.77. Finally, 123 patients (72.4%) were in the therapeutic INR target range of 2.0–3.5 (R-PT, 1.68–2.6; R-C, 1.78–2.89). The Pearson correlation coefficient r for the 170-patient sample was 0.78 (95% CI, 0.71–0.84; p < 0.001), and the Spearman correlation coefficient was 0.80 (95% CI, 0.73–0.85; p < 0.001). The linear equation is R-C = 1.0248 + (0.4425 × R-PT). Figure 1 shows the agreement between R-PT and R-C, with the differences between the two plotted against (R-C + R-PT)/2. As R-C values were lower than R-PT, the average difference was displaced 15.6% from 0.

### Correlation between concentration of factor X and clotting test results

A normal reference curve was created using the mean PPP values of four groups of normal volunteers (n = 5 in each group) measured on four consecutive days. Normal PPP was diluted with factor X-deficient plasma to obtain concentrations of factor X ranging from 1.56–100% (Table 1). In each dilution, PT, aPTT, DVVtest, and DVVconfirm were measured (in seconds) and expressed as a ratio (diluted PPP/undiluted PPP). TT was not assayed because different concentrations of factor X did not influence the clotting time using thrombin reagent.

**Table 1:** Influence of factor X concentration on clotting test results

	**Undiluted**	**Dilution**	**Dilution**	**Dilution**	**Dilution**	**Dilution**	**Dilution**	**p**
**Test**	**100%**	**50%**	**25%**	**12.50%**	**6.25%**	**3.13%**	**1.56%**	
R-PT	1	1.18 ± 0.07	1.42 ± 0.04	1.84 ± 0.14	2.09 ± 0.2	2.71 ± 0.08	3.44 ± 0.25	<0.001
R-aPTT	1	0.99 ± 0.14	1.08 ± 0.11	1.22 ± 0.08	1.3 ± 0.1	1.55 ± 0.26	1.8 ± 0.30	<0.001
R-VV	1	1.13 ± 0.07	1.36 ± 0.07	1.55 ± 0.08	1.85 ± 0.11	2.19 ± 0.24	2.73 ± 0.28	<0.001
R-C	1	1.17 ± 0.11	1.46 ± 0.14	1.79 ± 0.12	2.35 ± 0.16	3.11 ± 0.16	4.52 ± 0.33	<0.001
P		0.034	<0.001	<0.001	<0.001	<0.001	<0.001	

**Dilution 50**%**:** p < 0.05 for R-aPTT vs. R-PT, R-VV and R-C; **Dilution 25**%**:** p < 0.01 for R-aPTT vs. R-PT, R-VV, and R-C; **Dilution 12,5**%**:** p < 0.01 for R-aPTT vs. R-PT and R-C; p < 0.05 for R-aPTT vs. R-VV; p < 0.05 for R-VV vs. R-C and R-PT; **Dilution 6.25**%**:** p < 0.01 for R-C vs. R-aPTT; p < 0.05 for R-C vs. R-VV and R-PT; p < 0.01 for R-PT vs. R-aPTT; p < 0.05 for R-PT vs. R-VV; **Dilution 3.13**%**:** p < 0.01 for R-C vs. R-aPTT and R-VV; p < 0.05 for R-C vs. R-PT; p < 0.05 for RaPTT vs. R-VV and R-PT; **Dilution 1.56**%**:** p < 0.01 for R-C vs. R-aPTT, R-VV, and R-PT; p < 0.01 for R-aPTT vs. R-VV and R-PT.

R-C was the most sensitive to factor X concentration, followed by R-PT using Neoplastin Plus thromboplastin. R-VV had an intermediate sensibility, and R-aPTT was only slightly affected by factor X concentration.

### Rivaroxaban-treated patients

Twenty-five patients were treated with rivaroxaban. The dose was 20 mg/day for all patients, except one who took 15 mg/day. One patient was excluded because he also took rifampicin, which clearly prevented the effect of rivaroxaban on clotting parameters. Table 2 includes data for the 24 remaining patients.

**Table 2:** Anticoagulation effect of rivaroxaban in 24 patients, measured in PPP using different clotting assays

	Peak of activity* in hours					Trough of activity** in hours				
	**(mean ± SD, 2.06 ± 0.29)**					**(mean ± SD, 23.4 ± 1.09)**				
Ratio	R-PT	R-aPTT	R-TT	R-VV	R-C	R-PT	R-aPTT	R-TT	R-VV	R-C
Mean	1.72	1.32	1.15	3.08	3.53	1.20	1.08	1.11	1.66	1.89
SD	0.39	0.16	0.16	0.88	0.68	0.12	0.13	0.11	0.37	0.42
Median	1.61	1.28	1.11	2.75	3.53	1.17	1.03	1.2	1.62	1.97
Highest	2.53	1.74	1.67	5.13	5.63	1.46	1.38	1.26	2.35	2.88
Lowest	1.08	1.1	1.0	1.92	2.24	1.04	0.95	0.9	0.86	0.98

*p < 0.01 for R-VV vs. R-PT, R-aPTT, and R-TT; p < 0.01 for R-C vs. R-PT, R-aPTT, and R-TT.

**p < 0.05 for R-C vs. R-PT, R-aPTT, and RTT; p < 0.05 for R-VV vs. R-PT, R-aPTT, and RTT.

### Peak of activity

On average, R-PT was prolonged to 1.7 times the baseline value (1.72 ± 0.39), and R-aPTT was prolonged 1.3 times (1.32 ± 0.16). R-TT was slightly modified (1.15 ± 0.16). The most prolonged values were R-VV (3.08 ± 0.88) and R-C (3.53 ± 0.68).

### Trough of activity

On average, R-PT was prolonged to 1.2 times the baseline value (1.20 ± 0.12), and R- aPTT and R-TT were slightly modified (1.08 ± 0.13 and 1.11 ± 0.11, respectively). R-VV was prolonged more than 1.5 times (1.66 ± 0.37), and R-C was prolonged nearly 2 times (1.89 ± 0.42).

During both peak and trough activity, RVVtest and RVVconfirm were the most sensitive to rivaroxaban activity. aPTT and TT were only slightly affected by the anticoagulant capacity of rivaroxaban. In contrast to in the in vitro factor X concentration curves (Table 1), PT was found to be slightly modified.

## Discussion

An important issue relating to NOAC use is establishing the therapeutic range and assay used to monitor the anticoagulant effect. Clotting assays can be used to estimate the effects of different concentrations of NOACs spiked into normal plasma or in the plasma of patients taking the drugs. The first type of experiment does not account for the pharmacodynamics of the drug and potential drug interactions, and thus does not mirror the real situation in patients treated with NOACs. Moreover, studies in patients show that the drug concentrations vary during the day according to their activity peak and short half-life (peak and trough activities). The current study evaluated two references curves in comparison with the results obtained in patients taking rivaroxaban. A reference curve using the ratio (patient/normal) derived from the PT of 170 long-term stable patients treated with coumadins was compared with the ratio (R-C) obtained in the same patients using RVVconfirm reagent.

Some previous results suggest that the INR/ISI method is not suitable for measurement of rivaroxaban and dabigatran [10]. However, Harenberg et al. [33] reported that in ex vivo studies, STA Neoplastin Plus was the most precise method for determining the anticoagulant rivaroxaban in human plasma. Thus, we tested this reagent with a manufacturer-reported ISI of 1.31. PT is affected by the remaining activity of the so-called vitamin K-dependent factors, while R-C only indicates the remaining activity of factor X. In both assays, the end-point is thrombin generation. As mentioned by Brummel-Ziedins et al. [34], the thrombin generation profile is not dependent on only one factor, but on the synergy between all the factors. Therefore, slight differences in factor levels—all within the normal range—can lead to large differences in thrombin generation profiles, and there is unlikely to be a direct relationship between R-PT and R-C concerning the potential therapeutic and hemorrhagic level.

Normalized prothrombin times obtained with calibrated thromboplastin and instruments have improved patient safety in clinical practice. Maintaining INR values of ≥2.0 greatly reduces the incidence of stroke in patients with atrial fibrillation, and no increase of intracranial bleeding is observed with INR values of less than 4.0 [35]. These findings indicate that an INR between 2.0 and 3.5 (R-PT, 1.68–2.6) is a safe level for preventing stroke in patients with non-valvular atrial fibrillation, and R-PT was used as one measure for comparison in this study. R-PT and R-C showed a good correlation with a modest agreement; however, these results differed from those obtained in patients under rivaroxaban treatment. Patients with a mean R-PT of 1.72 had an R-C of 3.53 during peak rivaroxaban activity, and those with a mean R-PT of 1.2 during trough activity had an R-C of 1.89.

A purified fraction of Russell viper venom (RVV-X) converts FX to the active form of factor X (FXa) [36], forming the prothrombinase complex. The prothrombinase complex then activates prothrombin to thrombin, thus shortening the time of coagulation [37]. We hypothesized that a readily available reagent containing Russell’s viper venom (e.g., DVVreagent® and DVVconfirm®), which directly activates factor X to Factor Xa in the presence of phospholipids and calcium, could be used to assay residual thrombin in plasma after the effects of rivaroxaban on FXa, forming thrombin and probably other oral direct aFX. RVVconfirm has the advantage of not being influenced by heparin or lupus anticoagulant.

With this in mind, we tested a curve of different factor X concentrations using PT, aPTT, RVV, and RVVconfirm to compare the sensitivities of these assays and the levels of factor X. Our results indicated that RVVconfirm was most closely correlated with factor X concentration, followed by PT, and RVVtest, with aPTT being only slightly sensitive. Comparing these in vitro results with those obtained in patients under rivaroxaban therapy, we again found that RVVconfirm was the most sensitive, followed by RVVtest. Among the other clotting tests in patients treated with rivaroxaban, TT was not affected, aPTT was only slightly affected, and PT was barely affected. The present study did not aim to determine the drug concentration levels based on the degree of the anticoagulation effect in plasma of patients treated with rivaroxaban. However, Tripodi mentioned that the PT prolongations obtained in his patients were 1.54 times the baseline, corresponding to 215 ng/mL rivaroxaban in the plasma from patients taking 20 mg daily [21].

The current study performed assays in each patient at two different times: two hours after drug ingestion (peak activity), and close to the time of the next dose (trough activity). For oral anti-Xa drugs, TT was not sensitive, aPTT was not sensitive enough, and PT was prolonged but the degree of prolongation was highly variable with the thromboplastin used in our study. Tripodi et al. [38] showed that a different expression of PT results may be better suited for application in patients taking rivaroxaban.

It has been postulated that NOACs do not require routine laboratory monitoring due to their predictable pharmacokinetic and pharmacodynamic profiles. However, the current study results would indicate a need to monitor specific coagulation-related processes since R-C value close to the time of the next dose (trough activity) indicated no anticoagulant effect in one patient and probably excessive effect in other after 2 hours of drug ingestion. It is possible that the present results predict the therapeutic level in patients treated with rivaroxaban, and perhaps with other oral direct factor X inhibitors.

Several conclusions can be proposed based on hemorrhagic accidents in patients with congenital deficit of factor X. Among such patients, Brown and Kouides [39] proposed classification of severity based on FX:C activity measurements, with FX:C < 1% being severe, 1–5% being moderate, and 6–10% being mild. Bleeding is infrequent in patients with Factor X levels above 20%, while patients with FX levels < 10% present with mucocutaneous bleeding and those with moderate–severe deficiency may have symptoms, including hemarthrosis, intracranial hemorrhage, and gastrointestinal bleeding. These patients experience spontaneous bleeding when their plasma concentration of factor X is below 1% [40-43], and could also bleed with higher levels in a traumatic situation.

In the curves created with different Factor X concentrations, 1.56% factor X corresponded to an R-C of 4.52 ± 0.33, which we consider to be the measurement limit. None of the patients had renal dysfunction, but their clotting profile R-C varied between 5.63 and 2.24 at peak activity, and between 2.88 and 0.98 in the trough. Their R-VV profile varied between 5.13 and 1.92 at peak activity, and between 2.35 and 0.86 in the trough. In contrast, R-PT was slightly sensitive, and R-aPTT and R-TT were not sensitive enough to the effect of rivaroxaban. With the data obtained from the DVVconfirm test in 24 patients, one had an R-C above the theoretical point of safety (mean + SD; R-C 4.52 ± 0.33) at the peak of treatment. However, the efficacy limit cannot be deduced from the present study. It is also important to consider the safety data regarding the potential risk-benefit assessment of antithrombotic therapy, particularly in cases where dual and triple antithrombotic regimens are necessary in elderly patients [44].

One of the first concerns regarding NOACs was the lack of an antidote. Although an antidote for factor Xa inhibitors has now entered phase 2 studies in normal volunteers, there remains a need for an assay of drug activity. Our results in patients taking rivaroxaban demonstrated that at the activity peak where PT, aPTT, and TT were normal, R-VV and R-C were prolonged, likely indicating an adequate anticoagulation level. During the activity trough, most PT, aPTT, and TT values were normal or close to normal. In contrast, one patient showed no anticoagulant activity using RVVconfirm (R-V, 0.98), and low activity was indicated in three patients (R-C, 1.35, 1.47, and 1.58) whereas adequated anticoagulant effect were obtained in the other 20 patients. Similar results were obtained with RVVtest: three patients showed no anticoagulant effect (R-VV, 0.86, 1.1, and 1.16) and three patients exhibited likely low rivaroxaban activity (R-VV, 1.29, 1.32, and 1.38). Thus, it seems possible that R-C and R-VV can indicate the lack of rivaroxaban activity when drug use is discontinued due to episodes of uncontrolled bleeding during anticoagulation or for emergency surgery.

## Conclusions

In conclusion, we believe that dose adjustment based on therapeutic effect may be more appropriate than fixed-dose therapy but clinical trials are needed to support this recommendation. Laboratories should determine the sensitivity of their methods to the presence of NOAC. In spite of the available body of information, most current recommendations are based on the interpretations and opinions of experts.

It has been postulated that the rivaroxaban dose must be reduced in patients with impaired renal function according to creatinine clearance. However available assays do not account for drug interactions and, although creatinine clearance is easy to perform and Cockcroft-Gault equation can be used to estimate creatinine clearance for purposes of NOAC dosing the possible assay frequency depends on the patient’s clinical condition and adherence. Here we found that the R-C results were reasonably sensitive for rivaroxaban and likely also sensitive for other FX inhibitors. This assay can be performed rapidly, is not influenced by lupus anticoagulant and heparin, does not require a purified NOAC as a control, and measures whole-plasma clotting activity. Clinical studies are needed to establish definite cut-off values for R-C, and to determine whether they are useful for identifying patients at increased risk of hemorrhage or with low prevention effect. Additionally, patient adherence to therapy is of utmost importance.

### Addendum

A small group of patients were treated with dabigatran therapy (150 or 110 mg every 12 h; 4 patients in each group) using experimental conditions similar to those described in the current paper. The results suggest that DVVconfirm using R-C data is also sensitive to the anticoagulation effect of dabigatran.

## Competing interests

The authors declare that they have no competing interests.

## Authors' contributions

RA contributed to the conception, design, drafting and critical review of the manuscript and CDG contributed to the statistical analysis, drafting and critical review of the manuscript. Both authors have given their final approval.
